# Additive Effects of Exercise or Nutrition Intervention in a 24-Month Multidisciplinary Treatment with a Booster Intervention for Children and Adolescents with Overweight or Obesity: The ICAAN Study

**DOI:** 10.3390/nu14020387

**Published:** 2022-01-17

**Authors:** Sarah Woo, Young-Su Ju, Young-Gyun Seo, Yoon-Myung Kim, Hyunjung Lim, Kyung-Hee Park

**Affiliations:** 1 Department of Medical Sciences, College of Medicine, Hallym University, Chuncheon-si 24252, Korea; hjejcross@naver.com; 2 Department of Occupational Medicine, National Medical Center, Seoul 04564, Korea; zorro@nmc.or.kr; 3 Department of Family Medicine, Hallym University Sacred Heart Hospital, Hallym University, Anyang-si 14068, Korea; yg035@daum.net; 4 University College, Yonsei University International Campus, Incheon 21983, Korea; yoonkim@yonsei.ac.kr; 5 Department of Medical Nutrition, Kyung Hee University, Yongin-si 17104, Korea; hjlim@khu.ac.kr

**Keywords:** pediatric obesity, lifestyle intervention, booster intervention, maintenance therapy, BMI z-score, body composition, cardiometabolic risk markers

## Abstract

This study compared the effects of a real-world multidisciplinary intervention with additional exercise or nutritional elements and investigated the effectiveness of a booster intervention after weight regain. A total of 242 children and adolescents (age- and sex-specific body mass index [BMI] ≥ 85th percentile, mean age: 10.82 years, 60% male) were allocated to three groups: usual care, exercise, or nutrition. Six-month active treatment with 1:1 session and a maintenance stage with group activities were repeated twice to comprise a 24-month intervention. The primary outcome was change % of the BMI z-score (zBMI). A total of 110 (45.4%) participants completed the 24-month intervention. A mixed-effects model analysis indicated no significant interaction effect of the intervention group and treatment phase on the zBMI change % (*p* = 0.976). However, there was a significant main effect of the treatment phase on zBMI change % at 6 months (β = −2.98, [95% CI, −5.69–0.27]), 18 months (β = −3.99, [95% CI, −6.76–1.22]), and 24 months (β = −3.23, [95% CI, −5.94–0.52]; *p* = 0.042). The improvements in zBMI, body fat %, and cardiometabolic markers were observed only among males. Whereas the additive effect of intensive exercise or nutritional feedback was not detected in the long term, a booster intervention with 1:1 counseling was effective even after weight regain during the maintenance period. It may be useful to combine individualized counseling with a less intensive form of group care for long-term maintenance in a real-world setting.

## 1. Introduction

Childhood obesity is a health concern that requires immediate treatment, as children and adolescents with obesity are faced with a higher risk of diseases, such as metabolic syndrome, insulin resistance, non-alcoholic fatty liver disease, and dyslipidemia even before reaching adulthood [1,2]. The prevalence of pediatric obesity has become a global phenomenon, and although childhood obesity has been reported to level off in some economically developed countries, such as in Australia and France, most countries suffer from a wide prevalence of childhood obesity [3,4]. In Korea, the prevalence of childhood obesity has remained at 10–11.4% since 2010 [5], and public measures have taken place since 2005 to reduce its extent and minimize the health-related risks of childhood obesity [6]. As active therapies, such as pharmacotherapy and bariatric surgery, are often limited for children and adolescents, alternatively, lifestyle modifications have been widely used as the primary treatment for this age group [7].

Although the United States Preventive Services Taskforce (USPSTF) recommends a behavioral intervention of more than 26 contact hours for effective weight loss [8], intensive therapy is often not accessible to many patients in some regions. As such, an investigation of the effectiveness of a community-based intervention of lower intensity applicable in the real-world setting is needed [9,10].

Even after a successful intervention, a common problem is that weight regain occurs after treatment [11,12]. However, there is insufficient evidence related to the degree of weight regain according to the modality of the maintenance treatment for children and adolescents, e.g., group therapy versus individual counseling. Furthermore, adding booster sessions to a standard intervention is recommended as a beneficial strategy for long-term management to prevent regain among adults [13,14]. However, there is little evidence regarding the effects of a booster intervention after weight increase in children and adolescents.

Therefore, there is a need to determine the modality of lifestyle changing interventions applicable in the real world that have long-term effects on pediatric obesity. In the present study, we compared the effects of three long-term interventions for children and adolescents with different modalities, usual care, usual care with exercise training, and usual care with intensive nutritional education, on the body mass index (BMI) z-score (zBMI) change, body composition, and other metabolic markers. In addition, we aimed to explore the relationship between the treatment modality and weight change by implementing a booster intervention after the maintenance phase to determine the extent of weight regain and weight loss following weight regain.

## 2. Materials and Methods

### 2.1. Participants

A total of 242 children and adolescents aged 6–17 years (145 males, 97 females) participated in the Intervention for Childhood and Adolescent Obesity via Activity and Nutrition (ICAAN) study from 2017–2019, conducted at a university hospital in Korea. Children and adolescents with age- and sex-specific BMI ≥ 85th percentile, according to the 2007 Korean National Growth Charts [15], were included in the study. The recruitment was focused on participants with more than moderate obesity (age- and sex-specific BMI ≥ 95th percentile), and most of the participants (221, 91.3%) included had more than moderate obesity. Participants were excluded from the study if they were prescribed medications that can affect weight status, including corticosteroids, thyroid hormones, and insulin.

### 2.2. Study Design

**Figure 1:**
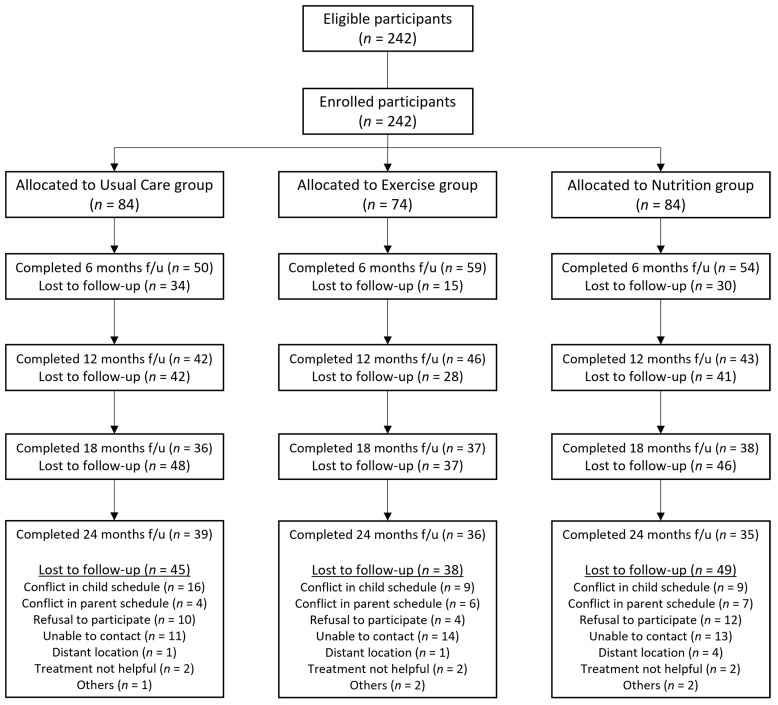
Flowchart of participant allocation.

The ICAAN study is a quasi-experimental trial designed to examine the effects of a 24-month lifestyle change intervention that can be replicated in a real-world setting. We aimed to compare the effects among three treatment groups: usual care, exercise, or nutrition. While the ICAAN study used a consecutive allocating procedure in assigning the participants, some participants who were unable to participate in the regular exercise programs were reassigned. For ethical purposes, these participants were not excluded from the study, to protect them from potential health risks due to lack of treatment. Figure 1 shows the flow of the allocation process and the follow-up rate.

This study was conducted in accordance with the guidelines of the Declaration of Helsinki and approved by the Institutional Review Board at Hallym University Sacred Heart Hospital (approval number: 2016-I135). Written informed consent was obtained from all participants and their primary caregivers. This study is registered at cris.nih.go.kr (identifier: KCT0002718).

### 2.3. Intervention

#### 2.3.1. Usual Care Group

**Figure 2:**
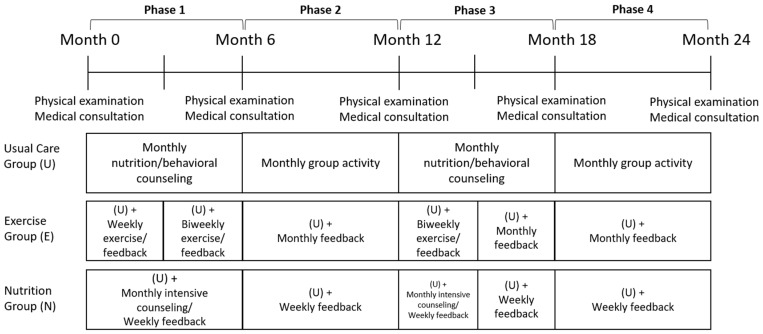
Flowchart of the intervention.

All participants received the usual care program consisting of four stages during the 24-month intervention, each lasting 6 months (Figure 2). Phases 1 and 3 involved active treatment, comprising 1:1 monthly counseling, and phases 2 and 4 were maintenance periods with a monthly group activity. A total of 10 nutrition education and behavioral counseling sessions, six group activities, six 1:1 counseling sessions, and four final group sessions were included in each phase. Health risk examination was conducted every 6 months, followed by consultation with a physician.

The behavior modification counseling employed cognitive behavior therapy strategies to practice problem-solving methods related to food intake and physical activity. Five main lifestyle goals, presented to the family as “Mission 5”, were reinforced at each session: screen time less than two hours, exercise more than one hour, eating more than five servings of fruits and vegetables, drinking plain milk or water instead of sugar-sweetened beverages, and more than eight hours of daily sleep. Nutrition education on portion size, food labels, and calorie intake was provided to promote the understanding of a healthy diet. To facilitate family support, parents were invited to a total of 10 sessions on parenting skills and provided with a monthly newsletter including exercise-related and nutritional information. An additional file describes the usual care program (see Supplementary Table S1). The total contact time of the usual care intervention was 19 h, which corresponds to low-intensity treatment resembling obesity treatment in the real-world setting.

#### 2.3.2. Exercise Group

In addition to the usual care, participants allocated to the exercise group attended exercise sessions. Individualized group exercise sessions were held weekly in the first 3 months of phase 1 and biweekly in the next 3 months of phase 1 and 3. Each session lasted 60 min, and the intensity of the exercise was at 60–80% of maximum heart rate (maxHR). The program consisted of six circuit training modules with 31 exercise movements (see Supplementary Table S2). Each module was performed for approximately 10 min (1 min for each movement with 30 s break), and every group exercise consisted of 3–4 modules. In addition to the group exercise, participants were recommended to perform 2–3 home-based exercises every week for 60 min per session, which consisted of 30 min of the circuit training exercise instructed during the group exercise, and 30 min of aerobic exercise including running or cycling. The home-based exercise was monitored with a daily exercise journal, and exercise education videos were handed out to all groups to encourage home-based exercise.

#### 2.3.3. Nutrition Group

In addition to the usual care, participants assigned to the nutrition group received 1:1 counseling based on the Nutritional Care Process (NCP) instead of the generalized education provided in the usual care program (see Supplementary Table S3) [16]. NCP is a systematic approach to provide individualized care through four main steps: nutrition assessment, diagnosis, intervention, and monitoring/evaluation [17]. In addition, to test for the effectiveness of a more frequent nutritional feedback, intensive feedback was provided through phone contact and text messages; weekly in the first 3 months, biweekly in the next 21 months.

### 2.4. Measurement

#### 2.4.1. Anthropometric Measurement

Anthropometric measurement was executed in the morning by a nurse after at least 8 h of fasting. Height was measured to the nearest 0.1 cm using a stadiometer, and weight was measured to the nearest 0.1 kg (InBody 720 Body Composition Analyzer, BioSpace Co., Ltd., Seoul, Korea). After the BMI score was calculated as weight (kg)/height (m^2^), zBMI was calculated based on the 2007 age- and sex-specific Korean National Growth Chart [15]. Body composition measurement was performed by a whole-body dual-energy X-ray absorptiometry (Lunar Prodigy Advance with pediatric software version enCore 14.0, GE Medical Systems Lunar). The fat mass index (FMI) and the fat-free mass index (FFMI) were calculated as the fat mass and fat-free mass (kg) divided by height squared (m^2^), respectively.

#### 2.4.2. Biochemical Assessment

Venous blood samples were collected by a nurse after at least 8 h of fasting. The levels (mg/dL) of high-density lipoprotein cholesterol (HDL-C) and low-density lipoprotein cholesterol (LDL-C) were measured using a homogeneous enzymatic colorimetric test. Triglyceride (TG; mg/dL), aspartate aminotransferase (AST; U/L), and alanine aminotransferase (ALT; U/L) were measured through an enzymatic assay. Fasting plasma glucose (mg/dL) was measured using an ultraviolet assay with hexokinase, while fasting plasma insulin (µU/mL) was measured using an electro-chemiluminescence immunoassay. All samples were processed with a Cobas 8000 C702 module (Roche, Mannheim, Germany). To specify the level of insulin resistance, the following formula indicating the homeostasis model assessment for insulin resistance (HOMA-IR) was used: [FBS (mg/dL) × fasting plasma insulin (μU/mL)]/405.

#### 2.4.3. Dietary and Physical Activity Assessment

Food intake was assessed through a 3-day food diary (2 weekdays, 1 weekend) including mealtimes and portion sizes. Each record was confirmed by a trained nutritionist during an individual interview. A web version of a nutritional analysis program (CAN Pro, 5.0; The Korean Nutrition Society, Seoul, Korea, 2016) was used to analyze the nutrient ingredients of the food records.

Physical activity was measured using the global physical activity questionnaire (GPAQ) [18]. The GPAQ comprises 16 items that evaluate physical activity in specific domains of daily activity, such as work and recreation, for which the Korean version has been validated [19].

### 2.5. Procedures

After the baseline measurement, follow-ups including anthropometric measurements and biochemical assessments were completed every 6 months thereafter. The zBMI change% from the baseline was the primary outcome of this study. Change from the baseline values of body composition variables (e.g., fat-free mass, fat mass, and fat%) and cardiometabolic risk markers (e.g., serum lipid, liver function, and insulin resistance) were the secondary outcomes.

### 2.6. Statistical Analysis

Baseline characteristics were compared among the three intervention groups. One-way analysis of variance with Bonferroni corrections and the Kruskal–Wallis test were performed for continuous variables with normal and non-normal distributions, respectively. Pearson’s chi-squared test was conducted for categorical variables. A linear mixed-effects model for repeated measures was used to analyze the interaction effect of the intervention groups with the treatment stages and the separate effects of the intervention groups and treatment stages on the primary and secondary outcomes. Participants who had completed the 24-months follow-up were included in the analysis. Sex, age, attendance rate, calorie intake, and physical activity (METS) were included in the model as covariates. A linear mixed model analysis with age, attendance rate, calorie intake, and physical activity (METS) as covariates was performed separately for males and females to analyze the effect of treatment stages on the outcome variables. The significance level was set at 0.05. The achieved power was 96% according to the number of participants recruited at baseline (242 participants). All statistical analyses were performed using SPSS 25.0 (IBM Corp., Armonk, NY, USA).

## 3. Results

### 3.1. Baseline Characteristics

The total participants’ and the intervention completers’ general characteristics and baseline laboratory test results are shown in Table 1. A total of 110 (45.5%) participants completed the 24-month intervention. The frequency of the total participants with overweight, moderate obesity, and severe obesity was 21 (8.7%), 143 (59.1%), and 78 (32.2%), respectively. Baseline characteristics did not differ significantly among the usual care, exercise, and nutrition groups. As the level of physical activity (METS) was significantly lower among the exercise group as compared to the nutrition group, it was included in the later analysis as a covariate to adjust for those differences. None of the baseline characteristics differed among intervention groups in treatment completers.

### 3.2. Changes in Primary Outcomes

**Figure 3:**
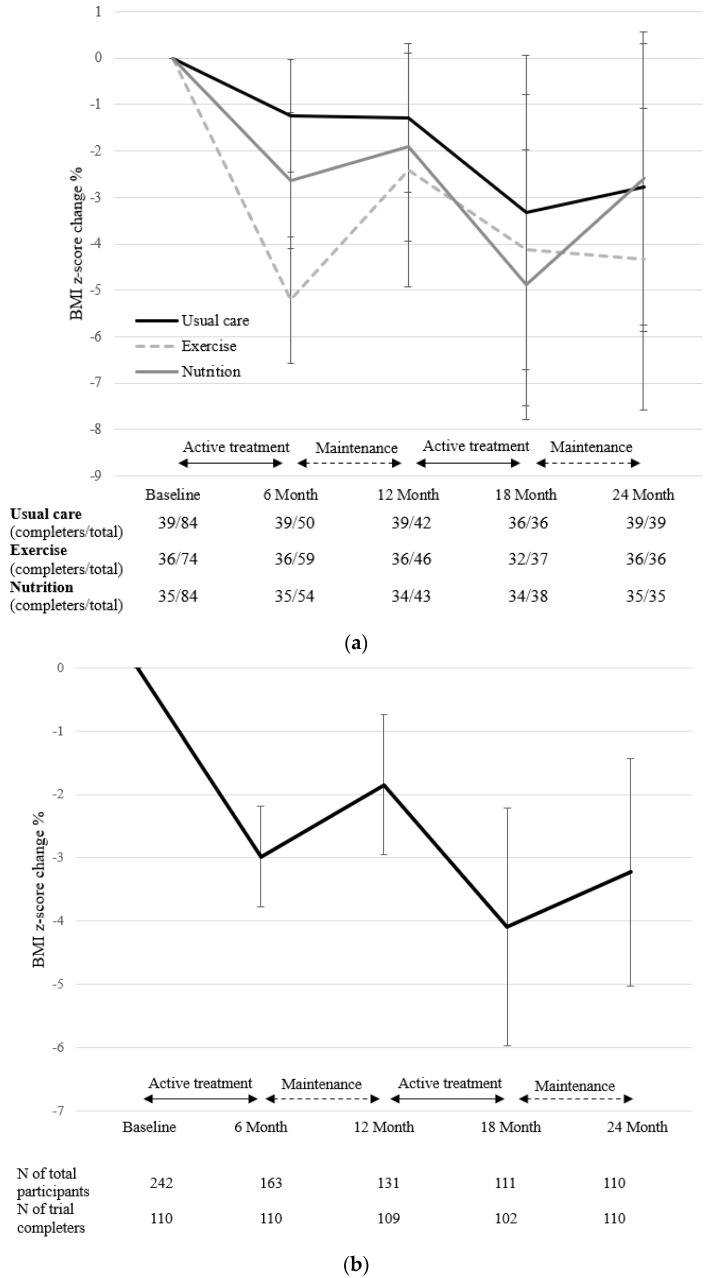
The course of BMI z-score change percentage from baseline according to the treatment stages. Linear mixed model adjusted for sex, age, attendance rate, calorie intake, and physical activity (METS). Figure shows means with standard error bars. (**a**) change % according to the intervention groups. Group effect (*p* = 0.772), time effect (*p* = 0.043), group × time interaction effect (*p* = 0.976). (**b**) change % in total, adjusted for intervention groups: time effect (*p* = 0.042).

The interaction effect of the intervention groups and treatment stages on the % change of the zBMI was not significant (*p* = 0.976), indicating that the % change of the zBMI did not differ among the intervention groups (Figure 3). The total effect of the treatment phases on the % change of the zBMI was significant (*p* = 0.042). The % change of the zBMI was significantly different from the baseline to 6 months (β = −2.98, [95% CI, −5.69, –0.27]), 18 months (β = −3.99, [95% CI. −6.76–1.22]), and 24 months (β = −3.23, [95% CI. −5.94–0.52), which are the active intervention, booster phase, and the second maintenance phase, respectively (Figure 3).

### 3.3. Changes in Secondary Outcomes

Table 2 shows that there was no significant interaction effect of the treatment phases and the intervention groups for ∆FMI (*p* = 0.753), ∆FFMI (*p* = 0.162), or ∆body fat % (*p* = 0.674). A test for the main effects revealed that the FFMI significantly increased across phases of the intervention (6 months: β = 0.22, [95% CI, 0.08–0.36], 12 months: β = 0.76, [95% CI, 0.62–0.90], 18 months: β = 1.04, [95% CI, 0.90–1.18], 24 months: β = 1.30, [95% CI, 1.16–1.44], *p* < 0.001). The FMI did not change significantly according to the treatment phases (*p* = 0.279), whereas the body fat % was highest at baseline and decreased afterward (6 months: β = −0.63, [95% CI, −1.24–0.02], 12 months: β = −1.21, [95% CI, −1.82–0.60], 18 months: β = −1.66, [95% CI, −2.29–1.03], 24 months: β = −1.74, [95% CI, −2.35–1.13], *p* < 0.001).

The liver function tests revealed that the ∆AST (*p* = 0.473) and ∆ALT (*p* = 0.398) levels did not differ among the treatment groups during the treatment course. The main effect of the treatment phases on the liver enzymes was significant, and AST (U/L) (*p* = 0.006) and ALT (U/L) (*p* < 0.001) decreased after 6 months (AST: β = −4.46, [95% CI, −7.23–1.69]; ALT: β = −16.87, [95% CI, −23.67–10.08]), 12 months (AST: β = −3.42, [95% CI, −6.21,–0.62]), 18 months (AST: β = −3.90, [95% CI, −6.76–1.05]; ALT: β = −12.67, [95% CI, −19.67–5.68]), and 24 months (AST: β = −4.79, [95% CI, −7.58–2.01]; ALT: β = −12.69, [95% CI, −19.52–5.86]).

The changes in TG levels did not differ among the treatment groups (*p* = 0.113), nor according to the treatment phases (*p* = 0.664). Meanwhile, ∆LDL-C levels changed according to the treatment phases and were significantly lower at 6 months (β = −4.87, [95% CI, −8.12–1.62]), 12 months (β = −4.52, [95% CI, −7.80–1.25]), and 24 months (β = −5.87, [95% CI, −9.14–2.60]) compared to those at baseline (*p* = 0.001). HDL-C increased at 12- (β = 1.82, [95% CI, 0.34–3.30]) and 18-months (β = 1.54, [95% CI, 0.03–3.05]) follow-up (*p* = 0.040).

HOMA-IR did not change significantly among the treatment groups (*p* = 0.940), while a deterioration was observed from baseline to 12 months (β = 0.97, [95% CI, 0.30–1.64]), 18 months (β = 1.00, [95% CI, 0.32–1.69]), and 24 months (β = 2.14, [95% CI, 1.47–2.81]).

### 3.4. Changes in Outcome Variables According to Sex

As sex was a significant covariate for zBMI change, males and females were analyzed separately. Table 3 shows that the changes in zBMI, body composition and cardiometabolic risk markers were most apparent among males but not females. Among males, FFMI increased (β = 0.38, [95% CI, 0.33–0.42], *p* < 0.001) while body fat % decreased (β = −0.64, [95% CI, −0.83–0.45], *p* < 0.001), with an overall zBMI decrease (β = −0.021, [95% CI, −0.037–0.005], *p* = 0.011). Meanwhile, among females, both FMI (β = 0.18, [95% CI, 0.09–0.26], *p* < 0.001) and FFMI (β = 0.28, [95% CI, 0.24–0.33], *p* < 0.001) increased with no significant change in either body fat or zBMI. Improvements in the cardiometabolic risk markers, including AST (β = −1.26, [95% CI, −2.20–0.31], *p* = 0.009) and LDL-C (β = −1.65, [95% CI, −2.53–0.77], *p* < 0.001) were detected only among males as well. None of the cardiometabolic markers improved among females, with the exception of an increase in HDL-C (β = 0.69, [95% CI, 0.13–1.25], *p* = 0.016). HOMA-IR significantly increased among both males (β = 0.39, [95% CI, 0.20–0.57], *p* < 0.001) and females (β = 0.63, [95% CI, 0.37–0.88], *p* < 0.001).

## 4. Discussion

The present study investigated the long-term effects of an additive exercise component and discussed the effectiveness of a booster intervention. This study demonstrated that whereas there was no long-term difference among the three intervention groups, the overall decline in the percent zBMI of the groups combined was significant in the long term. Although weight regain was observed during maintenance intervention, a booster intervention was effective in decreasing zBMI again after the maintenance period. However, the improvements in zBMI and cardiometabolic risk markers were found only among males.

According to previous research, the effectiveness of adding an intensive exercise component to a lifestyle intervention for children and adolescents is high for reducing BMI in the short term [20,21]. The short-term effects of adding an exercise program were significant in the ICAAN study as well, as the exercise group was the most successful in decreasing zBMI after 6 months of intervention, according to a previous paper on the ICAAN study [22]. Contrary to our expectations, the long-term effects of adding an exercise component to a multidisciplinary lifestyle intervention were not significantly different from the other groups who experienced more gradual weight loss. This is in line with previous studies that have reported an absence of long-term effects of an exercise intervention even when it was efficacious in the short term [23,24].

There are possible explanations for the absence of long-term effects of the additive exercise component. Whereas the structured and repetitive exercise protocol used in this study is known as an efficacious method in terms of weight loss [25], it may be difficult to maintain the interest to participate in the exercise protocol in the long term, especially for children and adolescents with obesity who may have difficulty in performing the exercise movements [26]. Adherence to an exercise protocol tends to be low (from 20% to 60%) among children and adolescents, even for short-term interventions [27,28]. Adherence by children and adolescents to the protocol of a long-term intervention is expected to be at an even lower rate. In addition, even when the supervised exercise sessions are effective in weight loss, weight could be regained if exercise is discontinued during the maintenance period when participants were encouraged to engage in unsupervised individual exercise [29]. In addition, individual factors, such as negative body image or mood, may have acted as barriers to benefiting from an exercise intervention [30].

The present study incorporated a design in which 1:1 active treatment (phase 1) and booster intervention (phase 3) were followed by a monthly group maintenance intervention (phase 2 and 4). The zBMI decreased at 6 and 18 months (phase 1 and 3), whereas weight regain was observed at 12 and 24 months (phase 2 and 4). As weight regain is commonly expected due to metabolic adaptations and changes in body composition following weight loss [31,32], a booster intervention may be necessary for long-term weight management to prevent further weight regain. A recent study comparing the effects of a 3-month intervention with or without a booster intervention for children reported that after 12 months, a decrease in zBMI was found only in the group with the booster intervention [33]. However, evidence is limited on the effectiveness of a booster intervention after weight regains for children and adolescents. The results of our study revealed that after weight regain, weight decreased again with a monthly booster intervention. Incorporating a periodical booster session post-intervention may be an effective method for long-term weight management applicable in the real-world setting.

In addition to a booster intervention, implementing a sustainable maintenance intervention with a lower intensity may be a means for preventing relapse during weight loss maintenance. According to a recent meta-analysis, the maintenance intervention was effective in stabilizing zBMI after weight loss [34], whereas weight was regained without it [35,36]. While a maintenance intervention seems necessary to prevent relapse following an intervention, evidence is inconclusive regarding the modality or the interval of successful maintenance. It has been reported that while a frequent intervention, such as a weekly individualized session, is more effective for further weight loss [37], a sporadic intervention, such as a monthly group meeting, can also result in the maintenance of weight loss [38]. The extent of the weight regain in our study was comparable to previous reports, with zBMI increases of 0.02 and 0.03 at 12 and 24 months, respectively. Overall, the monthly group-based maintenance intervention seems to be effective in stabilizing weight loss after an active intervention.

Among the secondary outcome variables, there were no differences between the intervention groups, but an overall improvement across treatment groups was observed. A long-term improvement was detected in zBMI, FFMI, body fat %, and cardiometabolic risk markers, such as ALT, AST, HDL-C, and LDL-C. Treatment outcome differed between the sexes; among males, an increase in FFMI with a decrease in body fat % led to a total decrease in zBMI. Improvements in cardiometabolic risk markers, such as AST and LDL-C, were observed only among males as well. Among females, however, both FMI and FFMI increased with a non-significant change in body fat % or zBMI. The sex differences in treatment outcomes may be related to the physiological changes during puberty among males and females [39]. Previous studies have reported a similar pattern of change in body composition according to sex [40,41], and our results indicate that sex differences are also present in changes in the cardiometabolic risk markers following an intervention. In addition to the biological differences, the lifestyle changes targeted in this study may have been more feasible for males than females. Males are known to participate in more physical activity than females [42], and previous studies have reported that parental engagement in restricting snacks is related to higher unrestricted eating and fat mass in females [43]. Therefore, sex difference should be taken into consideration when planning an obesity intervention for children and adolescents.

Meanwhile, adverse changes in HOMA-IR were observed for both sexes after the intervention, as it was highest at 24 months. This change may be the result of participants entering puberty during the intervention. Insulin sensitivity is reported to be the highest before the onset of puberty, to decrease during maturation, and to return to the original level at the end of the pubertal stage [44]. A less-intensive intervention on a monthly basis may be insufficient to ameliorate insulin resistance in children and adolescents, especially those who are going through the pubertal stage. As indicated in previous literature, an intensive intervention with a goal of reducing zBMI ≥ 0.5 may be needed to increase insulin sensitivity in children with obesity in the pubertal stage [45], and interventions with aerobic training components could be helpful in ameliorating insulin resistance [46].

There are some limitations to this study that require cautious interpretations of the findings. First, some participants who were unable to follow the protocol were reallocated; thus, complete randomization was not achieved. However, an analysis comparing the baseline characteristics did not reveal a significant difference among the three groups. Secondly, the dropout rate was relatively high at the end of treatment (54.5%). However, this is comparable to rates of other intervention studies on pediatric obesity, which vary widely, e.g., from 27–73% [47]. Additionally, a study of the factors related to dropouts in this study indicated that there were no systematic differences in the baseline variables between dropouts and completers [48]. Lastly, the overall zBMI decrease was 0.08, which is relatively small [8]. Whereas the majority of participants (59%) had a decrease in zBMI of more than 0.1, the overall decrease may seem smaller, as some participants did not respond to the treatment. Future research should investigate the factors associated with treatment non-response to account for the individual responses to treatment.

There are some significant strengths to this study. First, we compared the effects of a relatively less-intensive long-term intervention to the treatments with enhanced nutritional and exercise components. As participation in an intensive treatment is often not feasible for patients, the efficacy of a lower-intensity intervention designed to resemble weight-loss interventions in the real-world setting warrants investigation. Second, we examined the effectiveness of a booster intervention following weight regain. We observed that losing weight again is possible, even after experiencing weight regain post-intervention, with a booster intervention. Third, we compared the treatment outcomes according to sex and discovered that males in this age group are more responsive to a lifestyle intervention in terms of improvements in body composition and cardiometabolic risk markers. Sex-specific differences should be accounted for when anticipating outcomes of pediatric obesity interventions.

## 5. Conclusions

In conclusion, a monthly intervention may be both feasible and effective for long-term pediatric weight management. Given that it is difficult to conduct an intervention with the same modality for an extended period of time, incorporating a regular booster session during weight loss maintenance can be an effective strategy for long-term weight management in a real-world setting. Finally, as females may have disadvantages in benefiting from obesity intervention during the pubertal period, sex differences should be considered when planning an intervention for this age group.

## Figures and Tables

**Table 1:** Baseline demographic characteristics and measurements.

	Total Participants (*n* = 242)				Completers (*n* = 110)			
	Usual Care Group (*n* = 84)	Exercise Group (*n* = 74)	Nutrition Group (*n* = 84)	*p*-Value	Usual Care Group (*n* = 39)	Exercise Group (*n* = 36)	Nutrition Group (*n* = 35)	*p*-Value
Age *	11.02 (10.01, 12.09)	10.08 (9.04, 12.03)	11.01 (9.11, 12.05)	0.163	10.10 (9.10, 12.01)	10.05 (9.01, 11.76)	11.00 (9.06, 12.11)	0.461
Sex								
Male	51 (60.7%)	38 (51.4%)	56 (66.7%)	0.144	25 (64.1%)	18 (50.0%)	24 (68.6%)	0.243
Female	33 (39.3%)	36 (48.6%)	28 (33.3%)		14 (35.9%)	18 (50.0%)	11 (31.4%)	
BMI (kg/m^2^) *	27.65 (25.24, 30.13)	27.40 (25.08, 30.53)	27.65 (25.78, 30.85)	0.774	27.40 (24.60, 29.20)	27.50 (24.93, 30.48)	28.30 (25.30, 30.70)	0.549
BMI z-score *	2.15 (1.93, 2.55)	2.34 (2.01, 2.73)	2.23 (1.92, 2.59)	0.175	2.12 (1.94, 2.52)	2.37 (2.02, 2.73)	2.22 (1.98, 2.64)	0.322
Fat mass index *	11.30 (10.25, 12.93)	11.74 (9.83, 13.25)	11.45 (10.49, 13.29)	0.928	11.33 (10.30, 12.73)	11.08 (9.61, 13.23)	11.45 (10.54, 13.08)	0.660
Fat-free mass index *	15.72 (14.41, 17.67)	15.75 (14.72, 18.03)	16.27 (15.06, 17.61)	0.667	15.51 (14.40, 17.28)	15.73 (14.48, 17.65)	16.37 (14.55, 17.43)	0.464
Body fat %	41.87 ± 4.17	41.96 ± 4.51	41.83 ± 3.65	0.979	42.35 ± 3.87	41.48 ± 5.22	41.83 ± 3.16	0.661
Monthly household income								
<3 million KRW	8 (10.7%)	17 (23.0%)	13 (16.7%)	0.303	3 (8.1%)	2 (5.6%)	7 (20.6%)	0.272
3–5 million KRW	45 (60.0%)	37 (50.0%)	47 (60.3%)		22 (59.5%)	24 (66.7%)	20 (58.8%)	
≥5 million KRW	22 (29.3%)	20 (27.0%)	18 (23.1%)		12 (32.4%)	10 (27.8%)	7 (20.6%)	
AST (U/L) *	21.50 (18.00, 27.00)	22.00 (18.75, 27.25)	24.00 (19.00, 33.00)	0.117	21.00 (18.00, 28.00)	22.00 (18.00, 25.50)	25.00 (19.00, 33.00)	0.165
ALT (U/L) *	20.00 (14.00, 30.50)	21.50 (13.00, 2.35)	26.00 (16.00, 51.75)	0.053	19.00 (16.00, 29.00)	19.00 (13.00, 31.75)	26.00 (16.00, 53.00)	0.143
TG (mg/dL) *	99.00 (62.00, 147.50)	97.00 (80.00, 134.75)	103.00 (74.00, 137.75)	0.815	93.00 (67.00, 151.00)	101.50 (83.50, 144.00)	123.00 (68.00, 161.00)	0.623
HDL-C (mg/dL) *	50.00 (41.25, 59.00)	50.00 (41.75, 58.25)	48.50 (42.25, 57.00)	0.774	51.00 (42.00, 60.00)	47.00 (41.25, 53.75)	47.00 (40.00, 53.00)	0.279
LDL-C (mg/dL)	113.57 ± 23.64	110.49 ± 23.24	108.54 ± 25.96	0.403	118.03 ± 21.03	110.31 ± 23.44	110.60 ± 23.67	0.250
FBS (mg/dL) *	88.00 (82.25, 93.00)	88.00 (83.75, 94.00)	89.00 (95.00, 93.75)	0.424	89.00 (81.00, 93.00)	88.00 (84.00, 94.00)	89.00 (86.00, 93.00)	0.692
Insulin (μU/mL) *	16.80 (12.40, 23.48)	19.75 (13.08, 27.25)	19.20 (14.13, 26.25)	0.185	16.20 (12.40, 23.70)	20.30 (13.13, 25.25)	18.60 (14.20, 24.20)	0.578
HOMA-IR *	3.97 (2.64, 4.92)	4.09 (2.97, 6.23)	4.09 (3.00, 6.03)	0.140	3.63 (2.63, 5.05)	4.41 (2.92, 5.50)	4.09 (2.99, 5.61)	0.424
Total energy intake (kcal) *	2066.90 (1808.86, 2429.10)	2084.34 (1767.26, 2402.37)	2181.52 (1927.96, 2434.87)	0.234	2061.37 (1850.96, 2357.49)	1998.27 (1698.82, 2393.29)	2221.56 (1948.25, 2437.43)	0.267
Physical activity (METs) *	1146.00 (390.00, 4120.00)	1040.00 (390.00, 2530.00)	2240.00 (910.00, 4050.00)	0.014 †	1080.00 (480.00, 4000.00)	1080.00 (400.00, 2400.00)	2760.00 (600.00, 4560.00)	0.054

Abbreviations: BMI, body mass index; KRW, Korean Republic Won; AST, aspartate aminotransferase; ALT, alanine aminotransferase; TG, triglyceride; HDL-C, high-density lipoprotein cholesterol; LDL-C, low-density lipoprotein cholesterol; FBS, fasting blood sugar; HOMA-IR, homeostasis model assessment for insulin resistance; METs, metabolic equivalent of tasks. Data are presented as mean ± standard deviation for continuous variables (one-way ANOVA), and number (%) for categorical variables (chi-square test). * Median (interquartile range) for non-normally distributed data (Kruskal–Wallis test). † significant group difference between Exercise and Nutrition Group according to Bonferroni’s post hoc analysis.

**Table 2:** Changes in secondary outcomes according to intervention groups and treatment stages.

	Usual Care Group (*n* = 39)	Exercise Group (*n* = 36)	Nutrition Group (*n* = 35)	*p* ^a^	Total Participants (*n* = 110)	β (95% CI)	*p* ^b^
zBMI							
Baseline	2.27 (0.06)	2.39 (0.06)	2.27 (0.05)		2.31 (0.05)	reference	
change at 6 mo	−0.02 (0.05)	−0.12 (0.05)	−0.07 (0.05)		−0.07 (0.03)	−0.07 (−0.13, −0.02)	
change at 12 mo	−0.03 (0.05)	−0.06 (0.05)	−0.05 (0.05)		−0.04 (0.03)	−0.05 (−0.10, 0.01)	
change at 18 mo	−0.06 (0.05)	−0.10 (0.05)	−0.12 (0.05)		−0.09 (0.03)	−0.09 (−0.15, −0.04)	
change at 24 mo	−0.04 (0.05)	−0.09 (0.05)	−0.06 (0.05)	0.931	−0.07 (0.03)	−0.07 (−0.12, −0.01)	0.016
FMI							
Baseline	11.77 (0.36)	11.61 (0.48)	11.94 (0.41)		11.77 (0.24)	reference	
change at 6 mo	−0.01 (0.22)	−0.32 (0.24)	−0.01 (0.24)		−0.11 (0.14)	−0.14 (−0.42, 0.14)	
change at 12 mo	0.03 (0.22)	−0.01 (0.24)	−0.07 (0.24)		−0.02 (0.14)	−0.05 (−0.33, 0.24)	
change at 18 mo	0.12 (0.23)	0.19 (0.25)	−0.26 (0.25)		−0.02 (0.14)	−0.01 (−0.30, 0.28)	
change at 24 mo	0.20 (0.22)	0.27 (0.24)	0.15 (0.24)	0.753	0.21 (0.14)	0.18 (−0.11, 0.46)	0.279
FFMI							
Baseline	15.96 (0.39)	16.15 (0.37)	16.49 (0.37)		16.19 (0.22)	reference	
change at 6 mo	0.32 (0.11)	0.03 (0.12)	0.24 (0.12)		0.20 (0.07)	0.22 (0.08, 0.36)	
change at 12 mo	0.74 (0.11)	0.76 (0.12)	0.73 (0.12)		0.74 (0.07)	0.76 (0.62, 0.90)	
change at 18 mo	1.20 (0.11)	0.82 (0.12)	1.04 (0.12)		1.02 (0.07)	1.04 (0.90, 1.18)	
change at 24 mo	1.44 (0.11)	1.18 (0.11)	1.21 (0.12)	0.162	1.28 (0.07)	1.30 (1.16, 1.44)	<0.001
Body fat percentage (%)							
Baseline	42.35 (0.62)	41.48 (0.87)	41.83 (0.53)		41.90 (0.40)	reference	
change at 6 mo	−0.57 (0.49)	−0.63 (0.52)	−0.49 (0.53)		−0.56 (−1.14, 0.02)	−0.63 (−1.24, −0.02)	
change at 12 mo	−1.03 (0.49)	−1.21 (0.53)	−1.20 (0.53)		−1.14 (−1.72, −0.55)	−1.21 (−1.82, −0.60)	
change at 18 mo	−1.73 (0.51)	−0.91 (0.54)	−2.13 (0.55)		−1.60 (−2.20, −1.00)	−1.66 (−2.29, −1.03)	
change at 24 mo	−1.96 (0.49)	−1.37 (0.52)	−1.67 (0.53)	0.674	−1.68 (−2.26, −1.10)	−1.74 (−2.35, −1.13)	<0.001
AST (U/L)							
Baseline	27.51 (3.26)	25.39 (2.53)	32.40 (3.80)		28.37 (1.87)	reference	
change at 6 mo	−4.05 (2.55)	−0.98 (2.71)	−7.20 (2.73)		−4.09 (1.53)	−4.46 (−7.23, −1.69)	
change at 12 mo	−2.48 (2.57)	0.45 (2.71)	−7.19 (2.77)		−3.05 (1.55)	−3.42 (−6.21, −v0.62)	
change at 18 mo	−3.53 (2.63)	1.52 (2.82)	−8.26 (2.75)		−3.53 (1.57)	−3.90 (−6.76, −1.05)	
change at 24 mo	−3.34 (2.57)	−2.34 (2.73)	−7.66 (2.73)	0.473	−4.42 (1.54)	−4.79 (−7.58, −2.01)	0.006
ALT (U/L)							
Baseline	31.46 (6.41)	32.69 (6.21)	44.86 (7.60)		36.13 (3.90)	reference	
change at 6 mo	−7.59 (4.60)	−4.38 (4.87)	−14.94 (4.89)		−8.94 (2.76)	−16.87 (−23.67, −10.08)	
change at 12 mo	−0.73 (4.65)	7.31 (4.87)	0.49 (5.03)		2.21 (2.80)	−5.72 (−12.57, 1.13)	
change at 18 mo	−0.08 (4.83)	−1.47 (5.21)	−12.70 (4.95)		−4.74 (2.88)	−12.67 (−19.67, −5.68)	
change at 24 mo	−1.52 (4.65)	−3.36 (4.92)	−9.65 (4.89)	0.398	−4.76 (2.78)	−12.69 (−19.52, −5.86)	<0.001
TG (mg/dL)							
Baseline	110.13 (8.97)	114.22 (7.56)	118.63 (9.29)		114.17 (4.96)	reference	
change at 6 mo	6.15 (7.68)	10.21 (8.14)	−12.01 (8.20)		1.39 (4.62)	2.28 (−7.59, 12.14)	
change at 12 mo	15.58 (7.75)	−10.22 (8.14)	0.58 (8.38)		2.09 (4.67)	2.98 (−6.97, 12.93)	
change at 18 mo	3.55 (7.99)	−5.92 (8.59)	−13.13 (8.28)		−5.21 (4.78)	−4.33 (−14.49, 5.83)	
change at 24 mo	3.84 (7.75)	−5.03 (8.21)	−1.33 (8.20)	0.113	−0.88 (4.65)	0.00 (−9.92, 9.93)	0.664
HDL-C (mg/dL)							
Baseline	51.03 (1.66)	49.44 (2.06)	47.69 (1.63)		49.45 (1.03)	reference	
change at 6 mo	−0.26 (1.17)	−1.39 (1.24)	2.05 (1.25)		0.15 (0.70)	−0.03 (−1.49, 1.44)	
change at 12 mo	0.12 (1.18)	2.24 (1.24)	3.76 (1.28)		1.99 (0.71)	1.82 (0.34, 3.30)	
change at 18 mo	0.66 (1.22)	1.44 (1.31)	3.02 (1.26)		1.71 (0.73)	1.54 (0.03, 3.05)	
change at 24 mo	0.02 (1.18)	2.08 (1.25)	0.56 (1.25)	0.182	0.87 (0.71)	0.70 (−0.78, 2.17)	0.040
LDL-C (mg/dL)							
Baseline	118.03 (3.37)	110.31 (3.91)	110.60 (4.00)		113.14 (2.17)	reference	
change at 6 mo	−7.54 (2.54)	−3.47 (2.70)	−1.25 (2.71)		−4.15 (1.53)	−4.87 (−8.12, −1.62)	
change at 12 mo	−5.40 (2.57)	−3.38 (2.70)	−2.62 (2.77)		−3.80 (1.55)	−4.52 (−7.80, −1.25)	
change at 18 mo	−0.89 (2.64)	−2.08 (2.84)	1.31 (2.74)		−0.44 (1.58)	−1.17 (−4.52, 2.18)	
change at 24 mo	−8.35 (2.57)	−0.41 (2.72)	−6.42 (2.71)	0.233	−5.14 (1.54)	−5.87 (−9.14, −2.60)	0.001
HOMA-IR							
Baseline	4.18 (0.39)	4.89 (0.55)	4.44 (0.30)		4.49 (0.25)	reference	
change at 6 mo	0.41 (0.49)	0.05 (0.52)	0.87 (0.53)		0.44 (0.30)	0.39 (−0.28, 1.05)	
change at 12 mo	1.40 (0.50)	0.34 (0.52)	1.31 (0.54)		1.02 (0.30)	0.97 (0.30, 1.64)	
change at 18 mo	1.28 (0.52)	0.34 (0.56)	1.50 (0.53)		1.05 (0.31)	1.00 (0.32, 1.69)	
change at 24 mo	2.41 (0.50)	1.97 (0.53)	2.20 (0.53)	0.940	2.19 (0.30)	2.14 (1.47, 2.81)	<0.001

Abbreviations: BMI, body mass index; FMI, fat mass index; FFMI, fat-free mass index; AST, aspartate aminotransferase; ALT, alanine aminotransferase; TG, triglyceride; HDL-C, high-density lipoprotein cholesterol; LDL-C, low-density lipoprotein cholesterol; HOMA-IR, homeostasis model assessment for insulin resistance. Baseline values are presented as observed mean (standard error); change from baseline values are estimated mean (standard error) from the mixed effects linear regression model adjusted for sex, age, attendance rate, calorie intake, and physical activity (METS). *p* ^a^ group × time interaction effect; *p* ^b^ main effect of treatment phase.

**Table 3:** Changes in BMI z score, body composition, and cardiometabolic risk markers according to sex.

	Female (*n* = 43)		Male (*n* = 67)	
	Baseline	Post (24 Months)	Baseline	Post (24 Months)
BMI z score	2.43 (0.08)	2.39 (0.11)	2.23 (0.06)	2.16 (0.08) *
Body fat %	41.83 (0.66)	41.13 (0.72)	41.95 (0.50)	39.58 (0.75) ***
FMI	11.38 (0.36)	11.97 (0.47) ***	12.02 (0.32)	11.95 (0.38)
FFMI	15.71 (0.32)	16.77 (0.33) ***	16.50 (0.28)	17.94 (0.31) ***
AST (U/L)	24.74 (2.67)	23.88 (3.11)	30.70 (2.52)	23.09 (1.71) **
ALT (U/L)	26.81 (5.05)	26.98 (5.26)	42.10 (5.42)	32.60 (3.81)
TG (mg/dL)	120.58 (7.78)	114.33 (7.82)	110.06 (6.43)	115.20 (7.87)
HDL (mg/dL)	49.02 (1.76)	52.09 (1.84) *	49.72 (1.27)	48.91 (1.41)
LDL (mg/dL)	114.58 (2.82)	114.42 (4.36)	112.31 (2.85)	102.78 (2.93) ***
HOMA-IR	4.05 (0.24)	6.67 (0.81) ***	4.78 (0.37)	6.57 (0.47) ***

Abbreviations: BMI, body mass index; FMI, fat mass index; FFMI, fat-free mass index; AST, aspartate aminotransferase; ALT, alanine aminotransferase; TG, triglyceride; HDL-C, high-density lipoprotein cholesterol; LDL-C, low-density lipoprotein cholesterol; HOMA-IR, homeostasis model assessment for insulin resistance. Values presented as mean (standard error). Difference from baseline to post-treatment (mixed effects linear regression model adjusted for age, attendance rate, calorie intake, physical activity (METS), and intervention groups) * *p* < 0.05, ** *p* < 0.01, *** *p* < 0.001.

## Data Availability

The datasets used and/or analyzed during the current study are available from the corresponding author on reasonable request.

## Supplementary Materials

The following are available online at https://www.mdpi.com/article/10.3390/nu14020387/s1: Table S1: ICAAN program: Phase 1–4, Table S2: ICAAN Exercise Program, Table S3: ICAAN Nutritional Education Program.
