# Supplementary material for: Additive Effects of Exercise or Nutrition Intervention in a 24-Month Multidisciplinary Treatment with a Booster Intervention for Children and Adolescents with Overweight or Obesity: The ICAAN Study
Source: Nutrients. 2022 Jan 17;14(2):387. doi: 10.3390/nu14020387 (PMC8781150; doi:10.3390/nu14020387)
Supplement: Supplementary file 1 [file nutrients-14-00387-s001.zip › nutrients-1529589-supplementary/Supplementary material Table S1.pdf]

Supplementary Table S1. ICAAN program: Phase 1-4

| Session | Subject | Content |
|---|---|---|
| Phase 1 (1:1 intervention) | | |
| Month 1 | Learn my body | - Physical examination results<br>- Goal setting |
| Month 2 | Manage my body 1 | Self-examination<br>- Personal issues related to weight<br>- The environment: family and lifestyle |
| Month 3 | Manage my body 2 | Physical activity<br>- Activity level examination<br>- Individualized exercise plan |
| Month 4 | Manage my body 3 | Motivational enhancement and Mindfulness<br>- Encouraging health-related behavior choices<br>- Identifying problems and strengths |
| Month 5 | Manage my body 4 | Physical activity 2<br>- Increasing activity<br>- Decreasing inactivity |
| Month 6 | Manage my body 5 | Self-observation<br>- Acknowledging changes: body image<br>- Acknowledging changes: cognition |
| Phase 2 (group program) | | |
| Month 7 | Nutrition | Lose sugar and become super<br>Enjoy your food with all 5 senses |
| Month 8 | Physical activity | Zumba dance class |
| Month 9 | Behavioral modification | A journey to finding myself |
| Month 10 | Nutrition | Nutritional experimentation laboratory<br>Become a healthy grown-up |
| Month 11 | Physical activity | Pilates class |
| Month 12 | Behavioral modification | Stop and Think |
| Phase 3 (1:1 booster session) | | |
| Month 13 | Changes in action 1 | Self-evaluation of change<br>- Changes in the last 12 months<br>- Risk/protective factors |
| Month 14 | Changes in action 2 | Overcoming obstacles<br>- Motivational changes<br>- Resources for problem solving |
| Month 15 | Changes in action 3 | Finding strengths<br>- Validation of the problem-solving trials<br>- Encouraging individual strengths |
| Month 16 | Maintaining change 1 | Behavioral modification counseling<br>- Healthy habits to continue after treatment<br>- Expected difficulties and coping strategies |
| Month 17 | Maintaining change 2 | Nutritional counseling<br>- Healthy eating habits to continue after treatment<br>- Parental guide for weight loss maintenance |
| Month 18 | Maintaining change 3 | Exercise counseling<br>- Physical activities to continue after treatment<br>- Individualized goals after treatment termination |
| Phase 4 (group program) | | |
| Month 20 | Behavioral modification | Mindful eating: Learning my feelings and thoughts |
| Month 21 | Nutrition | What is in my refrigerator?<br>What can I eat in a convenience store? |
| Month 22 | Exercise | Tae-Bo (Taekwondo & Boxing) class |
| Month 23 | Behavioral modification | Learning to be on my own: making a resolution |
