# Supplementary material for: Additive Effects of Exercise or Nutrition Intervention in a 24-Month Multidisciplinary Treatment with a Booster Intervention for Children and Adolescents with Overweight or Obesity: The ICAAN Study
Source: Nutrients. 2022 Jan 17;14(2):387. doi: 10.3390/nu14020387 (PMC8781150; doi:10.3390/nu14020387)
Supplement: Supplementary file 1 [file nutrients-14-00387-s001.zip › nutrients-1529589-supplementary/Supplementary material Table S2.pdf]

Supplementary Table S2. ICAAN Exercise Program

Module	Exercise program
1	Butt Kicker → Wood Chop → Plank Jack → Squat → Lunge → Plank
2	Standing Bird Dog → Up-down Plank → Side Lunge → Rotational Chop → Running Mountain Climber → Plank
3	Alternating Fast Feet → In-and-Out Squat → Hands-up Side Bend → Side-to-Side Mountain Climber → Wall Sit → Plank
4	Jumping Jack → Buffy → Frogger Plank → Wall Squat → Side-Kick Mountain Climber → Plank
5	High Knee Run → Mountain Climber → Arm Walking → Jump Squat → Long Jump Buffy → Plank
6	Ski Jump → Wide Squat Pulse → Flow Push-up → Buffy Knee Kick → Scissors → Plank
