# Supplementary material for: Additive Effects of Exercise or Nutrition Intervention in a 24-Month Multidisciplinary Treatment with a Booster Intervention for Children and Adolescents with Overweight or Obesity: The ICAAN Study
Source: Nutrients. 2022 Jan 17;14(2):387. doi: 10.3390/nu14020387 (PMC8781150; doi:10.3390/nu14020387)
Supplement: Supplementary file 1 [file nutrients-14-00387-s001.zip › nutrients-1529589-supplementary/Supplementary material Table S3.pdf]

Supplementary Table S3. ICAAN Nutritional Education Program

Session	Usual care/Exercise Group	Nutrition Group
Phase 1		
Month 1	Checking the food diary<br>Nutrition education: A balanced diet	Individual management according to the Nutritional Care Process (NCP)<br>Checking the food diary<br>Nutrition education: A balanced diet
Month 2	Checking the food diary<br>Nutrition education: Healthy protein	Individual management according to the NCP<br>Checking the food diary<br>Nutrition education: Healthy protein
Month 3	Checking the food diary<br>Nutrition education: Five colors of fruits and vegetables	Individual management according to the NCP<br>Checking the food diary<br>Nutrition education: Five colors of fruits and vegetables
Month 4	Checking the food diary<br>Nutrition education: Food portions using hands	Individual management according to the NCP<br>Checking the food diary<br>Nutrition education: Food portions using hands
Month 5	Checking the food diary<br>Nutrition education: Learn the nutritional facts label	Individual management according to the NCP<br>Checking the food diary<br>Nutrition education: Learn the nutritional facts label
Month 6	Checking the food diary<br>Nutrition education: High calorie and low nutritional food	Individual management according to the NCP<br>Checking the food diary<br>Nutrition education: High calorie and low nutritional food
Phase 3		
Month 13	Checking the food diary<br>Problem solving/reinforcement according to the food intake monitoring	Individual management according to the NCP<br>Checking the food diary<br>Problem solving/reinforcement according to food intake monitoring
Month 14	Checking the food diary<br>Problem solving/reinforcement according to the food intake monitoring	Individual management according to the NCP<br>Checking the food diary<br>Problem solving/reinforcement according to food intake monitoring
Month 15	Checking the food diary<br>Problem solving/reinforcement according to the food intake monitoring	Individual management according to the NCP<br>Checking the food diary<br>Problem solving/reinforcement according to food intake monitoring
